# Nrf2 Mitigates RANKL and M-CSF Induced Osteoclast Differentiation via ROS-Dependent Mechanisms

**DOI:** 10.3390/antiox12122094

**Published:** 2023-12-10

**Authors:** Yang Yang, Zhiyuan Liu, Jinzhi Wu, Simeng Bao, Yanshuai Wang, Jiliang Li, Tao Song, Yongxin Sun, Jingbo Pi

**Affiliations:** 1 Department of Rehabilitation, The First Hospital of China Medical University, No. 155 Nanjing North Road, Shenyang 110001, China; yangyang@cmu.edu.cn; 2 Key Laboratory of Environmental Stress and Chronic Disease Control & Prevention, Ministry of Education, China Medical University, No. 77 Puhe Road, Shenyang 110122, China; zyliu@cmu.edu.cn (Z.L.); jzwu@cmu.edu.cn (J.W.); yswang@cmu.edu.cn (Y.W.); 3 Key Laboratory of Liaoning Province on Toxic and Biological Effects of Arsenic, China Medical University, No. 77 Puhe Road, Shenyang 110122, China; 4 Program of Environmental Toxicology, School of Public Health, China Medical University, No. 77 Puhe Road, Shenyang 110122, China; 5 Central Laboratory, Cancer Hospital of China Medical University, Cancer Hospital of Dalian University of Technology, Liaoning Cancer Hospital & Institute, No. 44 Xiaoheyan Road, Shenyang 110042, China; baosimeng@cancerhosp-ln-cmu.com; 6 Department of Biology, Indiana University Indianapolis, 723 West Michigan Street, SL 306, Indianapolis, IN 46202, USA; jilili@iupui.edu; 7 Department of Pain Medicine, The First Hospital of China Medical University, No. 155 Nanjing North Road, Shenyang 110001, China; 19911070@cmu.edu.cn

**Keywords:** Nrf2, ROS, c-FOS, osteoclast differentiation

## Abstract

Nuclear factor-erythroid 2-related factor 2 (Nrf2) has been shown to be a negative regulator of osteoclast differentiation, but the precise mechanisms have not yet been established. We examined the precise roles of Nrf2 in regulating antioxidants and reactive oxygen species (ROS) levels, especially the cytoplasmic and mitochondrial ROS during osteoclastogenesis in vitro. In the current study, we found that the absence of *Nrf2* promotes osteoclast differentiation in bone-marrow-derived macrophages (BMMs) and RAW 264.7 cells. The receptor activator of NF-κB ligand (RANKL) and macrophage colony-stimulating factor (M-CSF) significantly lowered the levels of Nrf2 and its downstream antioxidant enzymes at mRNA and/or protein levels during osteoclast differentiation in the BMMs of mice and RAW 264.7 mouse leukemic monocytes. Compared to the wild-type cells, *Nrf2-*deficient cells exhibited heightened sensitivity to both transient RANKL-induced cytoplasmic ROS and prolonged RANKL and M-CSF-induced cytoplasmic and mitochondrial ROS accumulation. Furthermore, exogenous antioxidant agents, including N-acetyl-cysteine (NAC), diphenyleneiodonium chloride (DPI), and mitoquinone mesylate (MitoQ), exhibited substantial capability to suppress the elevation of ROS levels during osteoclast differentiation induced by *Nrf2* deficiency, and they consequently inhibited osteoclast differentiation augmented by the lack of *Nrf2*. The activation of phosphorylated c-FOS resulting from elevated ROS promoted osteoclast differentiation. The inhibition of c-FOS blocked osteoclast differentiation, which was elevated by *Nrf2*-deficiency. Taken together, these data reveal that Nrf2 effectively decreased the accumulation of intracellular ROS and the phosphorylation of c-FOS during osteoclastic differentiation by regulating antioxidant enzymes and subsequently inhibited RANKL-induced osteoclast differentiation.

## 1. Introduction

Bone destruction is a major characteristic and severe consequence of multiple skeletal diseases, including osteoporosis and inflammatory arthritis. It significantly reduces the quality of life of these patients and increases their risk of disability [1,2]. Osteoporosis, a metabolic skeletal disease reflected by decreased bone mineral density, microarchitectural deterioration, and impaired bone strength, leads to an increased risk of fragility fracture. The development of osteoporosis and bone diseases is influenced significantly by aging, estrogen deficiency, and inflammation [3,4,5]. The fundamental etiology of osteoporosis involves an imbalance in bone turnover, characterized by a higher rate of bone resorption mediated by osteoclasts compared to the rate of bone formation mediated by osteoblasts.

Osteoclasts, which originate from osteoclast/macrophage/dendritic common progenitor cells, are multinuclear giant cells responsible for bone degradation. This process is achieved through the secretion of acid and proteolytic enzymes, including cathepsin K, which effectively dissolve collagen and other matrix proteins during bone resorption [6]. Osteoblasts secrete a receptor activator of nuclear factor-κB ligand (RANKL) that binds to the receptor activator of nuclear factor-κB (RANK) receptor on the surfaces of osteoclasts and their progenitors, thus promoting osteoclast differentiation and survival. The binding of RANKL and RANK recruits tumor necrosis factor receptor-associated factor 6 (TRAF6), which activates the downstream signaling of c-FOS and the nuclear factor of activated T cells, cytoplasmic 1 (NFATc1) [7]. c-FOS is a constituent of the FOS gene family and, in conjunction with Jun proteins, constitutes the AP-1 family of heterodimeric transcription factors. The interaction between c-FOS and the promoter region of NFATc1 leads to the activation of the NFATc1 gene [8], which is a key regulator of osteoclast-specific genes, including tartrate-resistant acid phosphatase (TRAP), H^+^-ATPase, Atp6v0d2, Oscar, and Cathepsin K marker genes [9]. The *c-Fos* knockout mice and transgenic mice that over-express dominant-negative c-JUN exhibited severe osteopetrosis.

Reactive oxygen species (ROS) are essential secondary intracellular messengers which mediate many of the molecular signaling pathways, including apoptosis, differentiation, and the activation of cell signaling cascades [10]. The involvement of free radicals and antioxidant systems in bone remodeling is of paramount importance [11]. It is plausible that natural antioxidants exert a protective influence on osteoporosis through the regulation of ROS levels [12]. Oxidative stress induced by ROS increases with age and/or estrogen deficiency in postmenopausal women, and can adversely affect bone homeostasis and lead to skeletal fragility [13,14]. In osteoclasts, ROS are important components that regulate the differentiation process [15,16]. The binding of RANKL to its receptor RANK leads to the activation of TRAF6, RAC1, and NADPH oxidase 1 (NOX1), resulting in an increase in intracellular ROS. Subsequently, the activation of MAPK, PI3K, and NF-κB signaling pathways occurs as a downstream event. ROS serves as the second messenger to facilitate the activation of these signaling pathways [17,18]. Mitochondrial ROS is also essential for hypoxia-induced osteoclast differentiation [19].

Nuclear factor erythroid 2-related factor 2 (Nrf2) is a transcription factor that is expressed in various cell types, such as osteoblasts, osteocytes, and osteoclasts [20]. It is increasingly acknowledged as a pivotal transcription factor that facilitates defense against electrophiles and oxidants, thereby promoting cell viability across multiple tissues [21]. Nrf2 exhibits binding affinity towards antioxidant response elements, thereby promoting the transcriptional activation of antioxidant proteins [22]. *Nrf2* deficiency leads to an increase in the intracellular ROS level, as well as a defect in the production of numerous antioxidant enzymes and glutathione, in both osteoclast precursors and osteoblast progenitor cells [23]. Exposure of RAW 264.7 macrophages to RANK ligands lowers the Nrf2/Keap1 ratio and leads to a decrease in the expression of Nrf2-dependent enzymes, which are in favor of ROS signaling [24]. *Nrf2* deficiency promotes the RANKL-induced activation of MAPK, including c-Jun N-terminal kinase, extracellular signal-regulated kinase, p38, and the consequent induction of NFATc1, a pivotal determinant of osteoclast differentiation [25,26]. Our previous studies found that *Nrf2* deficiency aggravates the increase in osteoclastogenesis and bone loss induced by inorganic arsenic [27]. Despite the extensive research conducted on the association between Nrf2 and osteoclast differentiation, further investigation is required in order to elucidate the precise mechanism by which Nrf2 influences the process of osteoclast differentiation.

While prior research suggests that ROS play a crucial role as secondary messengers in the differentiation of osteoclasts [15,16], there is a dearth of information concerning the impact of ROS on the population dynamics of osteoclasts in cells lacking *Nrf2*, particularly with respect to the contribution of c-FOS to the augmentation of osteoclast differentiation resulting from *Nrf2* deficiency. The underlying mechanisms by which *Nrf2* deficiency enhances osteoclastogenesis have not yet been fully elucidated. In this study, we examined the role of ROS and c-FOS in osteoclastogenesis in the absence of *Nrf2* using bone-marrow-derived macrophages (BMMs) and RAW 264.7 cells, and further identified the regulatory role of Nrf2 in c-FOS and NFATc1 activation.

## 2. Materials and Methods

### 2.1. Reagent

N-acetylcysteine (NAC, HY-B0215), diphenyleneiodonium chloride (DPI, HY-100965), mitoquinone mesylate (MitoQ, HY-100116A), blastcidin (HY-103401), and puromycin (HY-B1743A) were purchased from MedChemExpress (Shanghai, China). 2′,7′-dichlorofluorescein diacetate (DCFH-DA, S0033) and a BCA protein assay kit (P0027) were purchased from Beyotime Biotechnology (Shanghai, China). MitoSOX (M36008) was purchased from Thermo Fisher Scientific (Wilmington, NC, USA). Rhodamine phalloidin (KGMP001) and DAPI (KGR0001) were purchased from KeyGEN BioTECH (Nanjing, China). The mouse leukemic monocyte/macrophage cell line RAW 264.7 was purchased from American Type Culture Collection, ATCC (Manassas, VA, USA). Dulbecco’s modified essential media (DMEM), minimum essential medium alpha medium (α-MEM), fetal bovine serum (FBS), phosphate-buffered saline (PBS, pH 7.4), and supplements for cell culture were purchased from Life Technologies (Grand Island, NY, USA). Macrophage colony-stimulating factor (M-CSF, 416-ML-010) and RANKL (462-TEC-010) were purchased from R&D Systems (Minneapolis, MN, USA). Acid phosphatase kits (387A) were purchased from Sigma (St. Louis, MO, USA). Trizol reagent, SYBR mix, and iScript cDNA Supermix were purchased from TaKaRa (Dalian, China). The primers were designed using Primer Express (Applied Biosystems, Waltham, MA, USA) and synthesized by Life Technologies (Shanghai, China). Antibodies for NFATc1 (sc-7294), β-ACTIN (sc-47778), and p-c-FOS (sc-81485) were purchased from Santa Cruz Biotechnology (Santa Clara, CA, USA). Antibodies for GCLC (12601), GCLM (14241), and HO-1 (10701) were purchased from Proteintech (Rosemont, IL, USA).

### 2.2. Bone-Marrow-Derived Macrophages (BMMs) Extraction

*Nrf2*-wildtype (*Nrf2*^+/+^) and global *Nrf2* knockout (*Nrf2*^−/−^) littermate mice in C57BL/6 background were generated by crossing *Nrf2*-heterozygous (*Nrf2*^+/−^) mice, which were kindly provided by Dr. Masayuki Yamamoto (Tohoku University, Japan). Genotyping was performed using genomic DNA that was isolated from tail snips, as mentioned previously [27]. Mice were raised in a pathogen-free environment, with a diet of SPF grade and sterilized, purified drinking water. The room’s temperature was 23 ± 1 °C, the humidity was 55–70%, and a regular light/dark cycle was used (12 h day/12 h night). All animal experiments in this study were approved by the Institutional Animal Care and Use Committee of China Medical University (Shenyang, China), following all the current guidelines for animal care and welfare.

Cervical dislocation was used to euthanize the mice (8 weeks old, male), and they were immersed in 75% alcohol for 5 min for disinfection and sterilization. The bilateral femur and tibia were removed and collected. The long shaft was placed in a 4 mL centrifuge tube and transferred to a ventilation cabinet hood to ensure sterile operation for the remaining experiments. After the muscle and other tissues attached to long bones were carefully removed, epiphyses were cut off. Bone marrow was flushed out repeatedly using a 1 mL sterile syringe containing α-MEM culture medium until bone tissue turned white. The collected bone marrow was filtered using a 70 μm filter screen to remove the bone and muscle tissue residue in the culture medium. The sample was then centrifuged at 800 rpm for 5 min, and the supernatant was discarded. The bone marrow was re-suspended in 3 mL of culture medium and cultured in a 6 cm dish at 37 °C for 24 h. Then, the suspended cells were harvested and further cultured with 30 ng/mL M-CSF in α-MEM containing 10% (*v*/*v*) of heat-inactivated fetal bovine serum (HI-FBS). After 3 days of culture, the adherent cells were used for osteoclast differentiation studies.

### 2.3. Cell Culture

The mouse leukemic monocyte/macrophage cell line, RAW 264.7, was cultured in DMEM supplemented with 10% HI-FBS, 100 units/mL penicillin, 100 μg/mL streptomycin, and 10 mmol/L HEPES at 37 °C with 5% CO_2_. Scramble (Scr) and *Nrf2*-knockdown (*Nrf2*-KD) RAW 264.7 cells were established via MISSION shRNA lentiviral transduction (mouse Nrf2 (SHVRSNM_008686, Sigma, USA) or scrambled nontarget negative control (SHC002V, Sigma, USA) as described in [27]. The lentiviral transfer vectors encoding mouse *Nrf2* were developed as detailed previously [28]. The lentiviral transfer vectors encoding mouse *Nrf2* were constructed by cloning PCR-generated fragments into the lentiviral vector pLVX-IRES-NEO (PP2374, St. Louis, MO, USA) and pTK642, a gift from Dr. Tal Kafri of the University of North Carolina at Chapel Hill. Following transfection, the *Nrf2* overexpression *(Nrf2*-OE) cells were selected and maintained in culture medium containing 2 μg/mL blastcidin. In the current study, we define cells transfected with negative control lentiviral vectors as controls (Cont) in contrast to the *Nrf2*-OE.

### 2.4. TRAP Staining

Bone-marrow-derived OPCs (osteoclast precursor cells) and osteoclasts derived from RAW 264.7 cells were stained for the activity of TRAP to confirm the osteoclasts. The RAW 264.7 cell line was cultured in 24-well plates at a density of 1.5 × 10^4^ cells per well, and primary bone marrow hematopoietic stem cells were evenly distributed in 24-well plates at a density of 8 × 10^4^ cells per well. The culture medium, supplemented with 50 ng/mL RANKL and 30 ng/mL M-CSF, was refreshed every alternate day for a duration of 5 days. TRAP staining was then performed using an acid phosphatase kit according to the manufacturer’s instructions. The images of TRAP staining were captured using a microscope (DMi8, Lecia, Wetzlar, Germany). Analyses of the osteoclasts were implemented using ImageJ software (1.50i, NIH, Bethesda, MD, USA).

### 2.5. Quantitative Real-Time RT-PCR

Total RNA was extracted using the Trizol reagent following the manufacturer’s recommendations. Concentration of RNA was quantified by Nanodrop 2000 (Thermo, Wilmington, DE, USA). Reverse-transcription to cDNA (50 ng per sample) was carried out with iScript cDNA Supermix. Quantitative RT-PCR was performed using a reaction mixture containing SYBR mix, and real-time fluorescence was detected by QuantStudio 6 Flex (ABI, Foster City, CA, USA). The following sets of primers were used: *Cathepsin K* (5′-TGTATAACGCCACGGCAAA-3′ and 5′-GGTTCACATTATCACGGTCACA-3′), *H^+^-atpase* (5′-ACGGTGATGTCACAGCAGACGT-3′ and 5′-CCTCTGGATAGAGCCTGCCGCA-3′), *Apt6v0d2* (5′-GAAGCTGTCAACATTGCAGA-3′ and 5′-TCACCGTGATCCTTGCAGAAT-3′), and *Oscar* (5′-CTGCTGGTAACGGATCAGCTCCCCAGA-3′ and 5′-CCAAGGAGCCAGAACCTTCGAAACT-3′), which encode for proteins that are key to extracellular matrix degradation and bone resorption. *Nrf2* (5′-CGAGATATACGCAGGAGAGGTAAGA-3′ and 5′-GCTCGACAATGTTCTCCAGCTT-3′), Kelch-like ECH-associated protein-1, *Keap1* (5′-GTGGCCGTCACCATGGA-3′ and 5′-GCTTCAGCAGGTACAGTTTTGTTG-3′), glutamate cysteine ligase catalytic subunit, *Gclc* (5′-TGGCCACTATCTGCCCAATT-3′ and 5′-GTCTGACACGTAGCCTCGGTAA-3′), glutamate cysteine ligase modifier subunit, *Gclm* (5′-ACATTGAAGCCCAGGATTGG-3′ and 5′-CCCCTGCTCTTCACGATGAC-3′), Heme oxygenase-1, *Ho-1* (5′-CCTCACTGGCAGGAAATCATC-3′ and 5′-CCTCGTGGAGACGCTTTACATA-3′), NAD(P)H: quinine oxidoreductase-1, *Nqo1* (5′-TATCCTTCCGAGTCATCTCTAGCA-3′ and 5′-TCTGCAGCTTCCAGCTTCTTG-3′) were also used. *β-actin* (5′-GTATGACTCCACTCACGGCAAA-3′ and 5′-GGTCTCGCTCCTGGAAGATG-3′) was used as the internal control in all cases.

### 2.6. Flow Cytometry

Intracellular ROS levels were measured using a DCFH-DA or MitoSOX fluorescent probe. RAW 264.7 cells were harvested and washed twice with PBS. The cells were then incubated for 30 min in PBS containing 5 μM DCFH-DA or 3 μM MitoSOX. After being washed with PBS, the cells were suspended in PBS and intracellular ROS production was measured by flow cytometry (Becton Dickinson FACSCanto II, Becton Dickinson, San Jose, CA, USA) using excitation/emission wavelengths of 488/525 nm for DCFH-DA and 396/610 nm for MitoSOX. FlowJO software (10.8.1) was used to analyze ROS levels.

### 2.7. MitoSOX Red and DCFH-DA Staining

RAW 264.7 cells were grown in 24-well plates at a density of 1 × 10^4^/well overnight in the incubator until the cells reached 70–80%. The preparation of the DCFH-DA working solution occurred as follows: DCFH-DA and MitoSOX was diluted with serum-free medium for final concentrations of 5 μM or 4 μM, respectively. Serum-free medium, PBS, DCFH-DA, and MitoSOX working solution were pre-warmed in a 37 °C water bath before the experiment started; the 24-well plates containing cells were removed from the incubator and the medium was gently aspirated along the plate wall. The plate was washed twice with serum-free medium, gently, without touching the cells; next, 300 μL of DCFH-DA and/or MitoSOX working solution was added to each well. The incubation was continued at 37 °C for 30 min. The working solution was gently aspirated and washed twice with serum-free medium. Then, 300 μL of culture medium containing RANKL, NAC, DPI, or MitoQ was added to each well before being incubated again at 37 °C for 30 min. The treatment solution was discarded and washed once with PBS; 500 μL of PBS was added to each well again, and the cells were collected with a cell scraper and transferred into flow-through special glass tubes for the flow-through experiments.

### 2.8. Western Blot Analysis

RAW 264.7 cells were rinsed with pre-cooled PBS and lysed by 1× lysis buffer that contained 1 mM phenylmethylsulfonyl fluoride (PMSF). The protein concentration was quantified using a BCA protein assay kit. Next, 50 μg of protein was separated by SDS–PAGE and electrophoretically transferred to polyvinylidene difluoride (PVDF) membranes. The membranes were blocked with 3% BSA in Tris-buffered saline (TBS) containing 0.05% Tween-20 for 1 h. After blocking, the membranes were incubated with the corresponding primary antibodies overnight at 4 °C. NFATc1, GCLC, GCLM, HO-1, p-c-FOS, and β-ACTIN were used. Membranes were washed by 1× TBST, followed by incubation with anti-rabbit IgG-HRP for 1 h. Immunoreactive bands were visualized using Tanon 5500 (Tanon, Shanghai, China). The relative levels of the target proteins were normalized by β-ACTIN.

### 2.9. F-ACTIN Staining of Osteoclasts

RAW 264.7 cells were seeded in 6-well plates at 2 × 10^4^/well, then put back into the incubator to continue to culture for 24 h. The culture medium containing 50 ng/mL RANKL and 30 ng/mL M-CSF was changed every other day for 5 days. Cells were washed using PBS and fixed with 4% paraformaldehyde for 15 min, permeabilized in 0.1% Triton X-100 for 5 min, and subsequently stained with rhodamine phalloidin (20 min) and DAPI (5 min). After washing with PBS, representative images of F-ACTIN belt formation were captured using a fluorescence microscope (DMi8, Lecia, Germany).

### 2.10. Statistics

All experiments were repeated at least three times. The normality of the data was evaluated using the Shapiro–Wilk test, while the similarity between variances was tested using the Brown–Forsythe test. In cases where the data exhibited normal distribution and homogeneity of variance, Student’s *t*-tests were conducted for comparisons between two groups. For comparisons among multiple groups, a one-way or two-way ANOVA with Bonferroni post hoc tests was employed. The Kruskal–Wallis test was used for data with a non-normal distribution and/or non-homogeneity of variance, followed by Dunn’s multiple comparisons test. The data are represented as the mean (m) ± standard deviation (SD) values of independent replicates. All statistical analyses were performed using GraphPad Prism 5 (GraphPad Software, San Diego, CA, USA).

## 3. Results

### 3.1. Deficiency of Nrf2 Promotes the Differentiation of Osteoclasts

**Figure 1:**
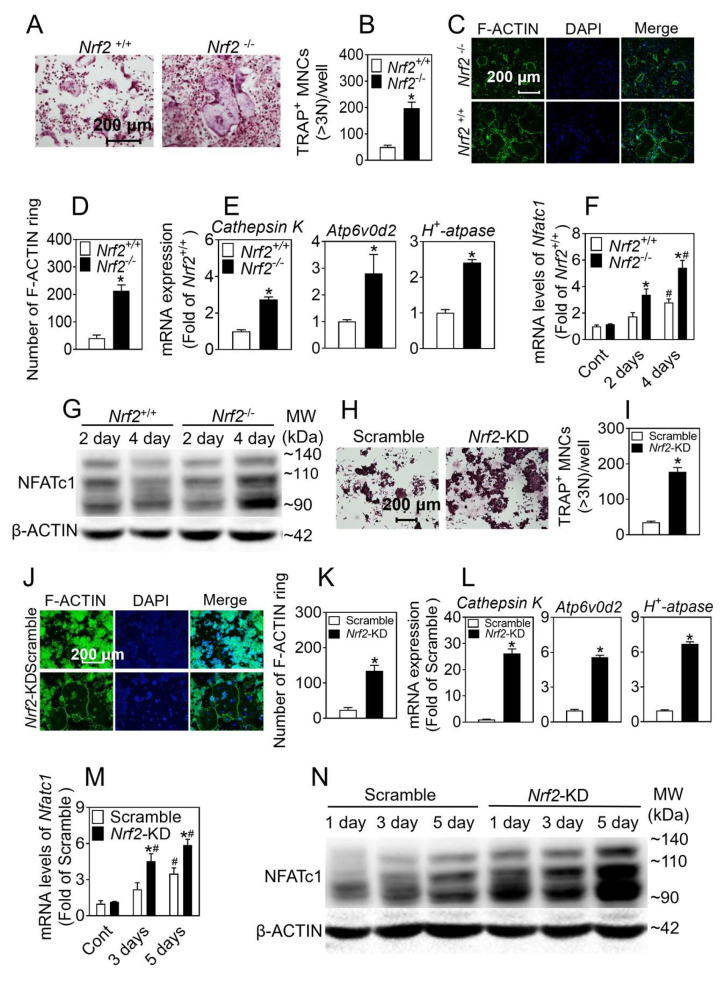
Deficiency of *Nrf2* promotes differentiation of osteoclasts. (**A**) Representative TRAP staining images of isolated BMMs treated with RANKL (50 ng/mL) and M-CSF (30 ng/mL) for 6 days. (**B**) Quantification of TRAP-positive multinucleated cells containing more than 3 nuclei. Staining (**C**) and quantification (**D**) of F-ACTIN ring formation of isolated BMMs. Green: F-ACTIN staining. Blue: DAPI staining. (**E**) mRNA levels of *Cathepsin K*, *Atp6v0d2,* and *H^+^*-*Atpase* in isolated BMMs treated with RANKL (50 ng/mL) and M-CSF (30 ng/mL) for 4 days. Values: mean ± SD. *n* = 3; Student’s *t* test was used; * *p* < 0.05 vs. *Nrf2^+/+^*. Scale bars = 200 μm. (**F**,**G**) mRNA and protein levels of NFATc1 of isolated BMMs treated with RANKL (50 ng/mL) and M-CSF (30 ng/mL) for 2 and 4 days. Values: mean ± SD. *n* = 3; two-way ANOVA was used; * *p* < 0.05 vs. *Nrf2*^+/+^ with the same treatment, # *p* < 0.05 vs. Cont of the same genotype. (**H**) TRAP staining of RAW 264.7 cells treated with RANKL (50 ng/mL) and M-CSF (30 ng/mL) for 4 days. Scale bars = 200 μm. (**I**) Quantification of TRAP-positive multinucleated cells containing more than 3 nuclei. (**J**,**K**) Staining and quantification of F-ACTIN ring formation of RAW 264.7 cells. (**L**) mRNA levels of *Cathepsin K*, *Atp6v0d2,* and *H^+^-Atpase* in *Nrf2*-KD RAW 264.7 cells treated with RANKL and M-CSF for 4 days. Values: mean ± SD. *n* = 3; Student’s *t* test was used; * *p* < 0.05 vs. scramble. Scale bars = 200 μm. (**M**,**N**) mRNA and protein levels of NFATc1 of *Nrf2*-KD RAW 264.7 cells treated with RANKL and M-CSF for 1, 3, and 5 days. Values: mean ± SD. *n* = 3; two-way ANOVA was used; * *p* < 0.05 vs. scramble with the same treatment; ^#^ *p* < 0.05 vs. Cont of the same cytotype.

*To* investigate the mechanisms underlying the abnormal osteoclast activation in *Nrf2*-deficient cells, we used osteoclast precursors from bone-marrow-derived cells and RAW 264.7 pre-osteoclast cells in the presence of M-CSF and RANKL to induce osteoclast differentiation. As shown in Figure 1A,B, following 5 days of induction, the formation of TRAP-positive multinucleated (≥3) cells containing more than three nuclei was significantly increased in *Nrf2^−/−^* cells. The *Nrf2*^−/−^ BMMs formed actin rings with clearer and denser margins than those formed in the *Nrf2*^+/+^ cells (Figure 1C,D). In addition, the *Nrf2^−/−^* cells also showed a high expression of *Cathepsin K*, *Atp6v0d2,* and *H^+^-atpase* (Figure 1E). We examined the expression of NFATc1 at day 2 and day 4 of culture, and found that the mRNA and protein levels of NFATc1 were also significantly higher in *Nrf2^−/−^* vs. *Nrf2^+/+^* (Figure 1F,G). As is consistent with these results, following induction to osteoclast differentiation, the knockdown of *Nrf2* in RAW 264.7 cells formed numerous TRAP-positive multinucleated osteoclasts; more F-ACTIN; higher mRNA levels of *Cathepsin K*, *Atp6v0d2, H^+^-Atpase,* and *Nfatc1*; and higher protein levels of NFATc1 (Figure 1H–N).

To further assess the role of Nrf2 in osteoclast differentiation, we developed a line of RAW 264.7 cells with stable overexpression of Nrf2. Compared with the control, *Nrf2*-OE cells showed high expression of mRNA and NRF2 protein (Figure S1A,B). Following the induction of osteoclast differentiation for 5 days, TRAP staining showed that the total number of osteoclasts and multinucleated osteoclasts possessing more than three nuclei were significantly reduced in *Nrf2*-OE cells (Figure S1C,D). Consistent with TRAP staining, the mRNA levels of *Cathepsin K, Atp6v0d2*, *H^+^-atpase,* and *Nfatc1* were decreased compared with the control (Figure S1E,F).

### 3.2. Nrf2 Exhibited a Decline during Osteoclast Differentiation, and Absence of Nrf2 Results in Decreased Expression of Antioxidant Enzymes and Increased ROS Levels

**Figure 2:**
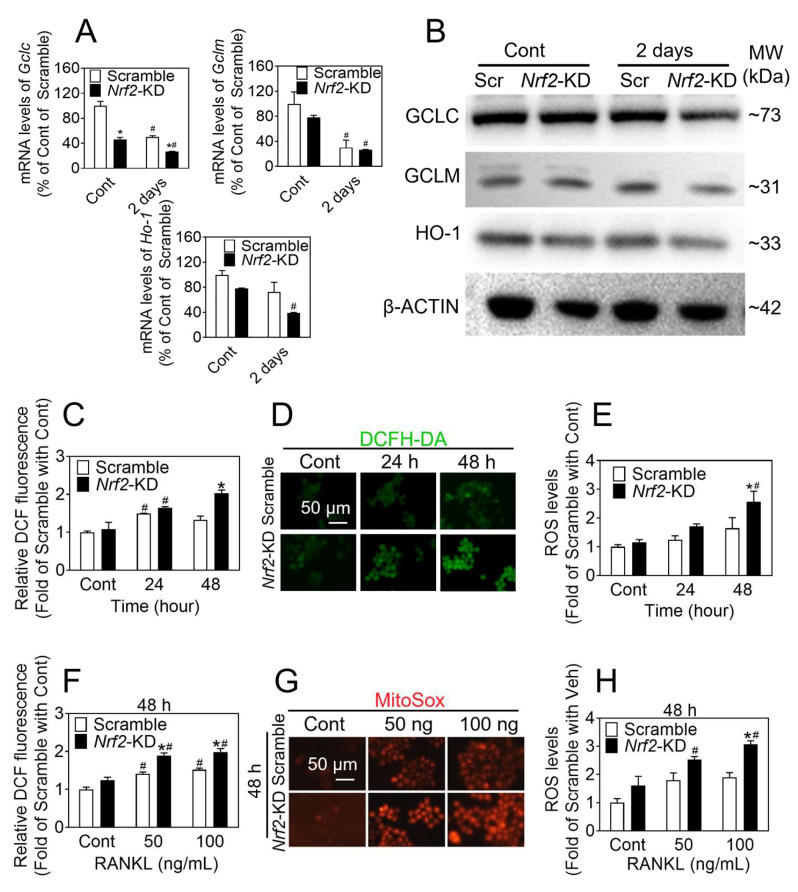
*Nrf2*-deficiency leads to elevated levels of ROS during osteoclast differentiation. Scramble and *Nrf2*-KD cells were treated with 50 ng/mL RANKL and 30 ng/mL M-CSF for 2 days, mRNA levels of *Gclc*, *Gclm,* and *Ho-1* were detected (**A**); protein levels of GCLC, GCLM, and HO-1 were detected (**B**). RAW 264.7 cells were treated with 50 ng/mL RANKL and 30 ng/mL M-CSF for 24 and 48 h, and then preloaded with DCFH-DA. ROS levels were measured by flow cytometry (**C**). Representative images and quantification of ROS levels measured by fluorescence microscopy (**D**,**E**). *Nrf2*-KD RAW 264.7 cells were treated with 50 and 100 ng/mL RANKL for 48 h and then preloaded with MitoSOX. ROS levels were measured by flow cytometry (**F**). Representative images and quantification of ROS levels measured by fluorescence microscopy (**G**,**H**). Scale bars: 50 μm. Values: mean ± SD. *n* = 3; two-way ANOVA was used; * *p* < 0.05 vs. scramble with the same treatment; ^#^ *p* < 0.05 vs. Cont of the same cytotype.

The expression level of antioxidative enzymes gradually decreased during osteoclast differentiation. To confirm the effect of Nrf2 signaling on antioxidant enzymes, we treated the BMMs and RAW 264.7 cells with 100 ng/mL RANKL for 0, 1, 2, 3, and 4 days, and found decreases in the gene expression levels of *Nrf2*, *Keap1,* and Nrf2-related antioxidant enzymes (*Nqo1*, *Ho-1*, *Gclc,* and *Gclm*) (Figure S2A,B). Consistent with these results, following induction to osteoclast differentiation for 2 days, the knockdown of *Nrf2* in RAW 264.7 cells formed led to antioxidant enzyme gene expression levels (Figure 2A,B).

ROS components are essential to the regulation of differentiation of osteoclasts, and have been found to be vital mediators regulating the differentiation of RANKL-stimulated osteoclasts. In order to further examine the changes in intracellular ROS level following RANKL treatment, we treated the RAW 264.7 cells with 50 and 100 ng/mL RANKL for 15 and 30 min and found that ROS levels peaked at 15 min after RANKL treatment, but declined thereafter (Figure S3A–C). As shown in Figure S3D–F, RANKL stimulation significantly increased intracellular ROS, demonstrating a dose–response relationship. To further determine whether RANKL-treated ROS production occurs in the cytoplasm or in the mitochondria, we used the DCFH-DA (ROS indicator fluorescent probe), MitoRed (to identify mitochondria), and MitoSOX (mitochondrial ROS probe), and found that RANKL-induced ROS generation is mainly concentrated in the cytoplasm (Figures S3 and S4).

We treated the RAW 264.7 cells with 100 ng/mL RANKL for 24 and 48 h; then, the intracellular ROS levels were measured by means of a confocal microscope and a flow cytometry assay. As shown in Figure 2C–E, in *Nrf2*-KD cells, intracellular ROS levels were markedly increased compared with the control group after RANKL treatment. ROS levels increased within 24 h, peaking at 48 h. We also treated RAW 264.7 cells with 50 and 100 ng/mL RANKL for 48 h, and the MitoSOX mitochondrial ROS probe results showed that ROS mainly concentrated in mitochondria (Figure 2F–H).

### 3.3. NAC and DPI Suppress Osteoclast Differentiation Augmented by Nrf2 Deficiency

In the preceding section of findings, it was observed that the administration of RANKL resulted in an immediate elevation in intracellular ROS. To elucidate the impact of this sudden rise in ROS on osteoclast differentiation, RAW 264.7 cells were subjected to a 15 min treatment of 100 ng/mL RANKL, with or without prior exposure to NAC (4 mM) or DPI (50 nM). The results indicate that pre-treatment with NAC and DPI led to a reduction in ROS levels (Figure S5A–D). RAW 264.7 cells were pretreated with NAC or DPI for 15 min and subsequently treated with 100 ng/mL RANKL and 50 ng/mL M^-^CSF for a period of 5 days. The number and size of TARP^+^ cells in *Nrf2*-knockdown (KD) cells were found to be significantly increased in comparison to scramble cells. However, the pretreatment with NAC and DPI did not exhibit any discernible effects on osteoclast differentiation (Figure S5E–G). The interim findings suggest that the temporary elevation of ROS triggered by RANKL administration did not impact the process of osteoclastogenesis.

**Figure 3:**
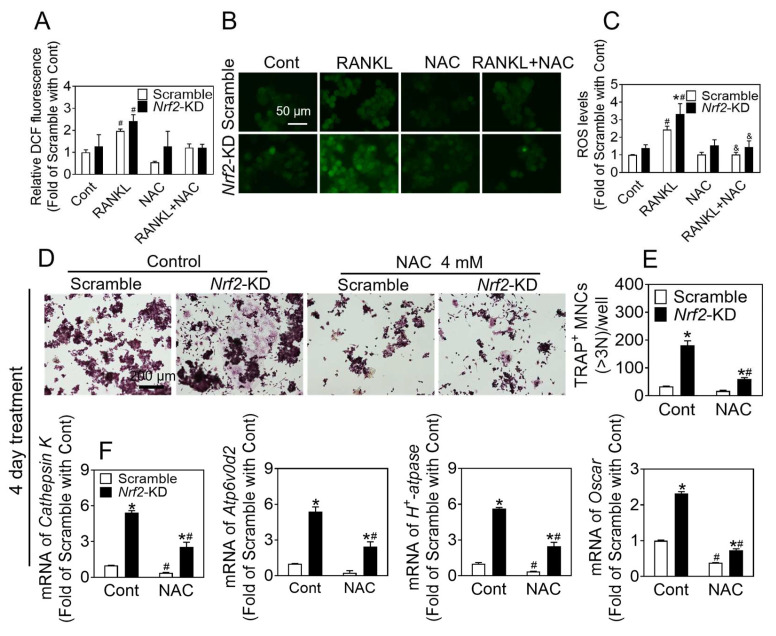
NAC treatment inhibits osteoclast differentiation induced by *Nrf2-*deficiency. (**A**–**C**) RAW 264.7 cells were treated with 50 ng/mL RANKL and 30 ng/mL M-CSF with or without NAC (4 mM) for 2 days, then preloaded with DCFH-DA. ROS levels were measured by flow cytometry (**A**). Representative images (**B**) of ROS levels were measured by fluorescence microscope, and quantification of ROS is shown (**C**). Scale bars: 50 μm. (**D**) TRAP staining of RAW 264.7 cells treated with RANKL (50 ng/mL) and M-CSF (30 ng/mL) for 4 days with or without of NAC (4 mM). (**E**) Quantification of TRAP-positive multinucleated cells containing more than 3 nuclei. Scale bars = 200 μm. (**F**) mRNA levels of *Cathepsin K*, *Atp6v0d2,* and *H^+^-Atpase* in *Nrf2*-KD RAW 264.7 cells treated with RANKL (50 ng/mL) and M-CSF (30 ng/mL) for 4 days, with or without NAC (4 mM). Values: mean ± SD. *n* = 3; two-way ANOVA was used; * *p* < 0.05 vs. scramble with the same treatment; ^#^ *p* < 0.05 vs. Cont of the same cytotype, ^&^ *p* < 0.05 vs. RANKL of the same cytotype.

To investigate the potential inhibitory effects of antioxidants on the promotion of osteoclast differentiation resulting from *Nrf2* deficiency, we subjected cells to treatment with 50 ng/mL RANKL, with or without NAC (4 mM), for a duration of 2 days. Our findings indicate that ROS levels were elevated in *Nrf2*-KD cells in comparison to scramble, and NAC effectively reduced ROS levels in both scramble and *Nrf2*-KD cells (Figure 3A–C). RAW 264.7 cells were exposed to a treatment consisting of 50 ng/mL RANKL and 30 ng/mL M-CSF for 4 days, with or without the presence of NAC (4 mM), during the initial 2 days. The results indicated a significant increase in the number and size of TARP^+^ cells in *Nrf2*-KD cells as compared to scramble cells. Furthermore, NAC was found to be effective in reducing osteoclast differentiation in *Nrf2*-KD cells, as depicted in Figure S6A–C. Consistently with the above results, RAW 264.7 cells were subjected to treatment with 50 ng/mL RANKL and 30 ng/mL M-CSF, in the presence or absence of NAC (4 mM), after a 4-day induction period. The administration of NAC was observed to effectively inhibit osteoclast differentiation (Figure 3D–F).

**Figure 4:**
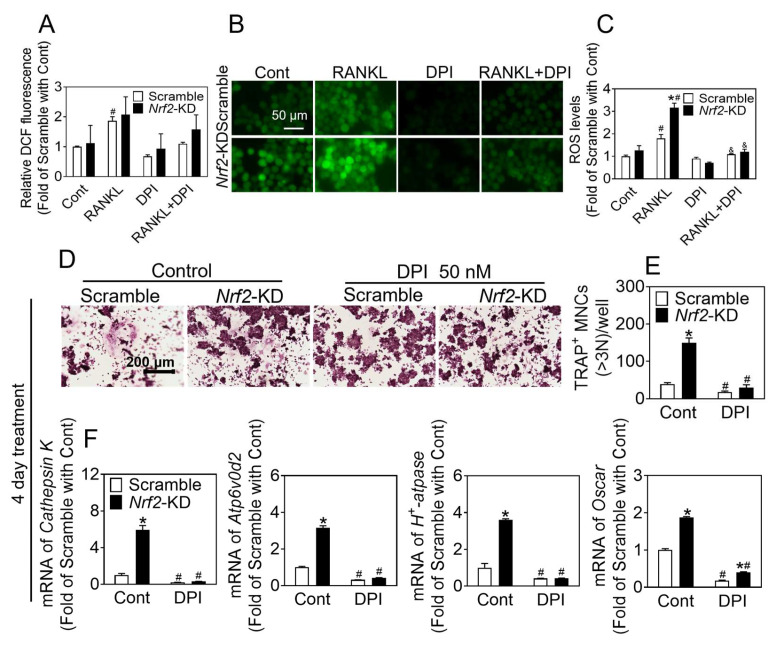
DPI treatment inhibits osteoclast differentiation induced by *Nrf2-*deficiency. (**A**–**C**) RAW 264.7 cells were treated with 50 ng/mL RANKL and 30 ng/mL M-CSF, with or without DPI (50 nM), for 2 days, then preloaded with DCFH-DA. ROS levels were measured by flow cytometry (**A**). Representative images (**B**) of ROS levels were measured by a fluorescence microscope, and quantification of ROS is shown (**C**). Scale bars = 50 μm. (**D**) TRAP staining of RAW 264.7 cells treated with RANKL (50 ng/mL) and M-CSF (30 ng/mL) for 4 days with or without of DPI (50 nM). (**E**) Quantification of TRAP-positive multinucleated cells containing more than 3 nuclei. Scale bars = 200 μm. (**F**) mRNA levels of *Cathepsin K*, *Atp6v0d2*, and *H^+^-Atpase* in *Nrf2*-KD RAW 264.7 cells treated with RANKL (50 ng/mL) and M-CSF (30 ng/mL) for 4 days with or without of DPI (50 nM). Values: mean ± SD. *n* = 3; two-way ANOVA was used; * *p* < 0.05 vs. scramble with the same treatment; ^#^ *p* < 0.05 vs. Cont of the same cytotype. ^&^ *p* < 0.05 vs. RANKL of the same cytotype.

In order to further substantiate the inhibitory effects of antioxidants on the promotion of osteoclast differentiation resulting from *Nrf2* deficiency, we administered a treatment of 50 ng/mL RANKL, with or without DPI (50 nM), for a duration of 2 days. Our findings indicate that there was a significant increase in ROS levels in *Nrf2*-KD cells as compared to scramble cells, and that DPI effectively reduced ROS levels in both scramble and *Nrf2*-KD cells (Figure 4A–C). Additionally, RAW 264.7 cells were subjected to treatment with RANKL (50 ng/mL) and M-CSF (30 ng/mL), with or without DPI, for a period of 2 or 4 days, as illustrated in Figure S7 and Figure 4D–F. DPI exhibited the ability to impede osteoclast differentiation as well as to suppress the promotion of osteoclast differentiation resulting from *Nrf2* deficiency.

### 3.4. MitoQ Suppresses Enhanced Osteoclast Differentiation Augmented by Nrf2 Deficiency

**Figure 5:**
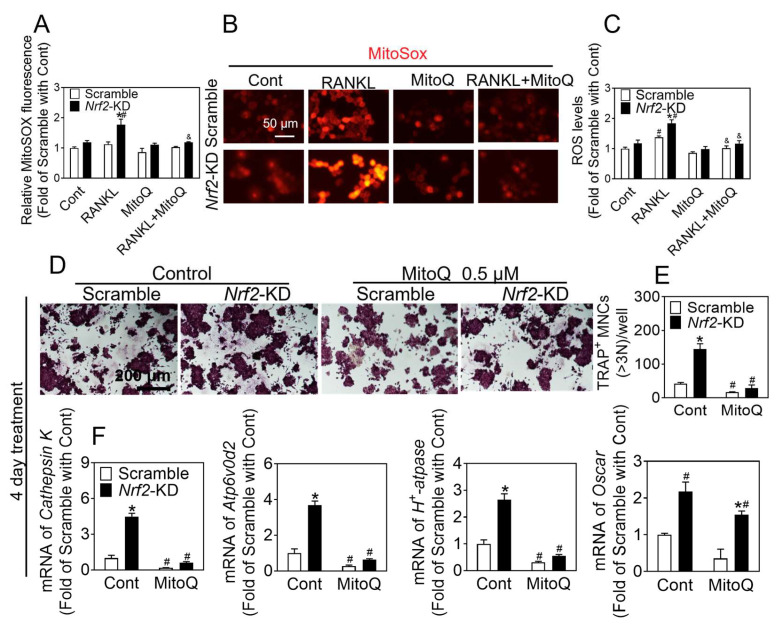
MitoQ treatment inhibits osteoclast differentiation induced by *Nrf2-*deficiency. (**A**–**C**) RAW 264.7 cells were treated with 50 ng/mL RANKL and 30 ng/mL M-CSF with or without MitoQ (0.5 μM) for 2 days, and then were preloaded with MitoSOX. ROS levels were measured by flow cytometry (**A**). Representative images of ROS levels were measured by fluorescence microscopy (**B**), and quantification of ROS is shown (**C**). Scale bars = 50 μm. (**D**) TRAP staining of RAW 264.7 cells treated with RANKL (50 ng/mL) and M-CSF (30 ng/mL) for 4 days, with or without MitoQ (0.5 μM). Scale bars = 200 μm. (**E**) Quantification of TRAP-positive multinucleated cells containing more than 3 nuclei. (**F**) mRNA levels of *Cathepsin K*, *Atp6v0d2,* and *H^+^-Atpase* in *Nrf2*-KD RAW 264.7 cells treated with RANKL (50 ng/mL) and M-CSF (30 ng/mL) for 4 days with or without of MitoQ (0.5 μM). Values: mean ± SD. *n* = 3; two-way ANOVA was used; * *p* < 0.05 vs. scramble with the same treatment; ^#^ *p* < 0.05 vs. Cont of the same cytotype. ^&^ *p* < 0.05 vs. RANKL of the same cytotype.

In the preceding findings, it was observed that exposure to RANKL resulted in heightened levels of ROS within the mitochondria. To assess the impact of mitochondrial ROS production on osteoclast differentiation, we treated the RAW 264.7 cells with RANKL with or without MitoQ (mitochondria-specific ROS scavenger) for 2 days. As illustrated in Figure 5A–C, the administration of MitoQ resulted in a decrease in reactive ROS levels in control cells and effectively prevented any elevation in ROS content in *Nrf2*-KD cells. Furthermore, co-treatment of RANKL (50 ng/mL), M-CSF (30 ng/mL), and MitoQ for 2 and 4 days inhibited the formation of osteoclasts and downregulated several genes associated with osteoclastogenesis (Figure S8 and Figure 5D–F). These data were consistent with the findings shown above. *Nrf2*-deficiency consistently resulted in an increase in ROS triggered by RANKL, thereby stimulating the differentiation of osteoclasts.

### 3.5. Inhibition of c-FOS Blocks Osteoclast Differentiation Elevated by Nrf2 Deficiency

**Figure 6:**
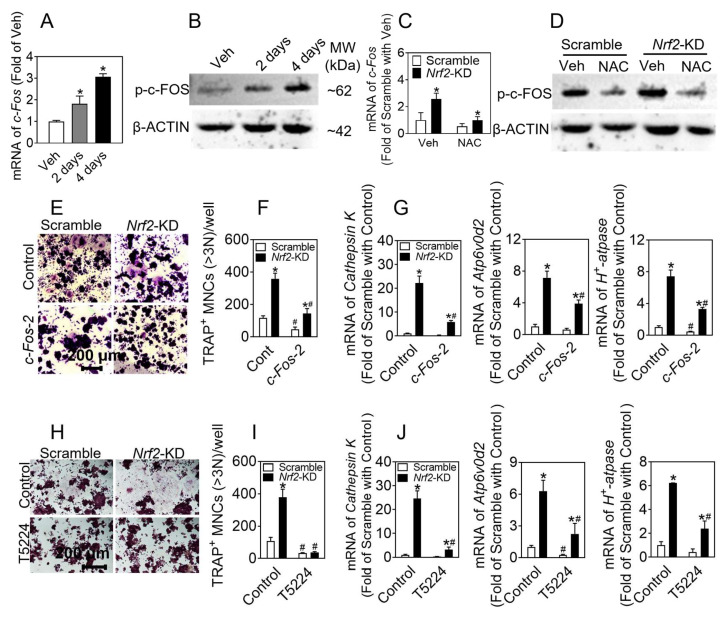
Inhibition of phosphorylation of c-FOS blocks osteoclast differentiation**.** (**A**,**B**) mRNA and protein levels of c-FOS in RAW 264.7 cells treated with RANKL (50 ng/mL) and M-CSF (30 ng/mL) for 2 and 4 days. Values: mean ± SD. *n* = 3; one-way ANOVA was used; * *p* < 0.05 vs. Veh. Veh, vehicle. (**C**,**D**) Expression of c*-Fos* mRNA and p-c-FOS protein in *Nrf2*-KD RAW 264.7 cells treated with RANKL (50 ng/mL) and M-CSF (30 ng/mL) for 2 days, along with NAC (4 mM). Values: mean ± SD. *n* = 3; two-way ANOVA was used; * *p* < 0.05 vs. scramble with the same treatment. (**E**–**G**) RAW 264.7 cells were treated with 50 ng/mL RANKL and 30 ng/mL M-CSF for 4 days. TRAP staining of RAW 264.7 cells (**E**). Quantification of TRAP-positive multinucleated cells containing more than 3 nuclei (**F**). Scale bars = 200 μm. (**G**) mRNA levels of *Cathepsin K*, *Atp6v0d2,* and *H^+^-Atpase* in *Nrf2*-KD and *Nrf2/c-F*os *double* KD cells. (**H**,**I**) TRAP staining of *Nrf2*-KD RAW 264.7 cells treated with RANKL (50 ng/mL) and M-CSF (30 ng/mL), with or without T5224 (4 μM), for 4 days. TRAP staining of RAW 264.7 cells (**H**). Quantification of TRAP-positive multinucleated cells containing more than 3 nuclei (**I**). Scale bars = 200 μm. (**J**) mRNA expression of *Cathepsin K*, *Atp6v0d2,* and *H^+^-Atpase* in *Nrf2*-KD RAW 264.7 cells. Values: mean ± SD. *n* = 3; two-way ANOVA was used; * *p* < 0.05 vs. scramble with the same treatment; ^#^ *p* < 0.05 vs. Cont of the same cytotype.

Previous studies have shown that c-FOS is crucial for osteoclast differentiation, which can upregulate osteoclast marker genes, such as *Oscar*, *Trap*, *Atp6v0d2*, and *Cathepsin K,* during osteoclastogenesis. To investigate the role of c-FOS in osteoclast differentiation in *Nrf2*-deficiency cells, we detected the mRNA and protein levels of c-FOS during osteoclast differentiation and found that the expression of c-FOS was significantly increased, and in *Nrf2*-KD cells, the expression of c-FOS was significantly higher compared with the control (Figure 6A–D).

To determine the role of c-FOS in the enhancement of osteoclast differentiation caused by *Nrf2* deficiency, we developed a line of RAW 264.7 cells with stable knockdown of *c-Fos* in scramble and *Nrf2*-KD cells. Compared with the RAW 264.7 cells expressing non-specific control shRNA (control), *c-Fos-1* and *c-Fos-2* knockdown cells had significantly reduced mRNA and protein expression of c-Fos in both scramble and *Nrf2*-KD cells (Figure S9A–D). The *c-Fos-2* knockdown cells, which exhibited the most evident knockdown efficacy, were selected for follow-up experiments. The levels of NFATc1 were also decreased in *c-Fos* knockdown cells (Figure S9B,D). Following 5 days of induction, the number of TARP^+^ (nuclear ≥ 3) cells in *Nrf2*-KD cells substantially increased compared to scramble cells, and *c-Fos* knockdown significantly inhibited osteoclast differentiation, especially in *Nrf2*-KD cells (Figure 6E,F). The mRNA levels of *Cathepsin K*, *Atp6v0d2,* and *H^+^-atpase* were also reduced in *c-Fos* knockdown cells (Figure 6G).

To elucidate the impact of c-FOS on osteoclast differentiation elevated by *Nrf2*-deficiency, we treated RAW 264.7 cells with T5224 (10–100 μM) for 24 h and found that T5224 (50 μM) can significantly suppress c-FOS and NFATc1 expression (Figure S10). Therefore, 50 μM T5224 was chosen for the subsequent experiments. Scramble and *Nrf2*-KD cells were treated with RANKL (50 ng/mL) and M-CSF (30 ng/mL), with or without T5224 (50 μM), to induce osteoclast differentiation. The number of TARP^+^ (nuclear ≥ 3) osteoclasts in *Nrf2*-KD was much higher than in scramble at day 5, and T5224 significantly reduced the number of osteoclasts, especially in *Nrf2*-KD cells (Figure 6H,I). Likewise, the mRNA of *Cathepsin K*, *Atp6v0d2,* and *H^+^-atpase* showed the same results (Figure 6J).

## 4. Discussion

**Figure 7:**
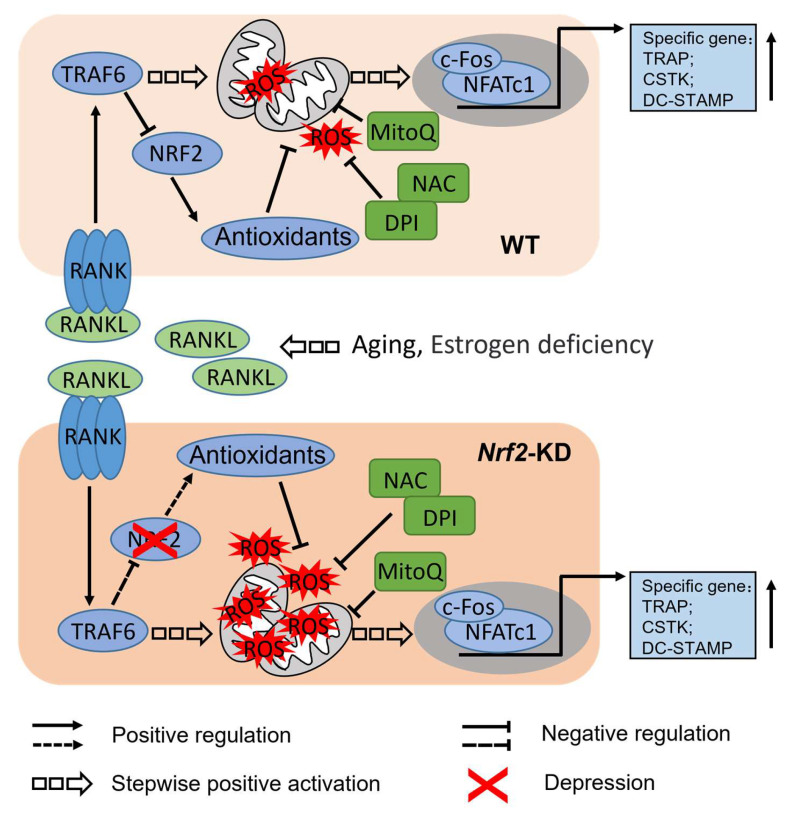
Schematic illustration of the proposed mechanism of Nrf2 in osteoporosis. During the process of differentiation, the modulation of antioxidant enzyme expression can be achieved by suppressing the expression of Nrf2 in cells. This suppression subsequently results in an elevation in intracellular ROS levels and activation of the c-FOS/NFATc1 signaling pathway, thereby facilitating osteoclast differentiation. Notably, the inhibition of intracellular ROS levels and c-FOS phosphorylation can effectively impede the augmented osteoclast differentiation induced by *Nrf2* deletion. This finding suggests that Nrf2 possesses the ability to hinder c-FOS expression by suppressing cellular ROS levels, thereby exerting an inhibitory effect on differentiation. Arrow and dotted arrow represent positive regulation, Flat-headed arrow and Flat-headed dotted arrow represent negative regulation, hollow dotted arrow represents stepwise positive activation and red “X” represents depression.

Numerous reports have demonstrated a significant association between oxidative stress and osteoporosis in the general population. However, the precise mechanism underlying this relationship, particularly the impact of advanced age and estrogen deficiency on osteoclast differentiation, remains elusive. While a substantial body of literature suggests that ROS may serve as a secondary messenger in osteoclast differentiation, further investigation is required in order to elucidate the role of abnormally elevated ROS in individuals with osteoporosis. Consequently, this study aims to examine the influence of ROS on osteoclast differentiation. In this study, we found that a deficiency of *Nrf2* in BMMs and RAW 264.7 cells resulted in elevated osteoclast differentiation, where overexpression of *Nrf2* in RAW 264.7 cells inhibited osteoclast differentiation. Analysis of the intracellular ROS level in response to RANKL treatment revealed that the administration of RANKL quickly resulted in a peak of ROS levels within the cytoplasm at the 15 min mark. The treatment of RANKL for 24 and 48 h led to an elevation in both cytoplasmic and mitochondrial ROS levels. *Nrf2* deficiency increased the sensitivity of BMMs and RAW 264.7 to RANKL-induced ROS production. Treatment with ROS inhibitors such as NAC, DPI, and MitoQ significantly blocked osteoclast differentiation, which was elevated by *Nrf2* deficiency. During the process of osteoclast differentiation, an upregulation of c-FOS expression was noted, with a more pronounced effect observed in cells lacking *Nrf2.* Additionally, the reduction in c-FOS levels hindered the process of osteoclastic differentiation. Moreover, it was discovered that antioxidants effectively suppressed the abnormal elevation of c-FOS levels induced by *Nrf2* deficiency, subsequently impeding osteoclast differentiation (Figure 7). The data presented in this study offer compelling evidence that Nrf2 plays a crucial role in osteoclast differentiation through the modulation of ROS and c-FOS.

Nrf2, a redox-sensitive transcription factor, plays a crucial role in providing adaptive protection against oxidative stress through the activation of various cytoprotective genes. Nrf2 has been found to be linked to age-related bone loss, specifically senile osteoporosis, as well as postmenopausal osteoporosis. The administration of the Nrf2 agonist dimethyl fumarate has been observed to impede the progression of osteoporosis in ovariectomized mice [29]. Consequently, an imbalance in this mechanism leads to an elevation in oxidative stress and a decrease in Nrf2 levels. Previous studies have demonstrated that the absence of *Nrf2* contributes to the differentiation of osteoclasts induced by oxidative stress. Therefore, in order to mitigate the development of osteoporosis by reducing the levels of ROS, the Nrf2-mediated regulation of antioxidant enzymes such as *Ho-1*, *Nqo1*, *Gclc*, and *Gclm* should be taken into account [30]. During osteoclast differentiation, our study revealed that treatment with RANKL and M-CSF resulted in a notable decrease in Nrf2 expression. Furthermore, the absence of *Nrf2* in both RAW 264.7 cells and BMMs augmented the osteoclast differentiation induced by RANKL and M-CSF. Conversely, the overexpression of *Nrf2* in RAW 264.7 cells hindered the osteoclast differentiation. Additionally, our findings demonstrated that the deficiency of *Nrf2* during osteoclast differentiation led to a reduction in the expression levels of antioxidant enzymes. Moreover, the levels of ROS in the cytoplasm and mitochondria of osteoclasts increased, with a more pronounced increase observed in osteoclasts lacking *Nrf2.* The above results suggest that osteoclast differentiation can be promoted by decreasing intracellular Nrf2 levels, and when *Nrf2* is absent, the level of antioxidant enzymes mediated by *Nrf2* decreases, leading to enhanced osteoclast differentiation.

Oxidative stress frequently occurs as a pathological condition resulting from estrogen deficiency, aging, hyperglycemia, and hyperlipemia [31]. The redox state changes are also related to the bone remodeling process, which allows for continuous bone regeneration [32,33]. In osteoclast differentiation, redundant ROS in cells can trigger many disorders, including inflammation, aging, metabolic disturbance, and osteoporosis. Oxidative stress can activate the differentiation of pre-osteoclasts to osteoclasts and increase bone resorption [34,35]. The regulation of redox status during osteoclast differentiation plays an important role in maintaining bone homeostasis [36]. A plethora of natural plant active compounds have been found to be capable of inhibiting osteoclast-specific marker genes, including transcription factors such as c-FOS, NFATc1, and c-Src. These compounds also counteract the effects of local factors, such as ROS and NO, while suppressing the activation of diverse signaling pathways, such as MAPK and NF-κB, thereby impeding osteoclast differentiation [31]. Mitochondrial ROS are essential for the hypoxic enhancement of osteoclast differentiation. In osteoclast differentiation, TRAF6 plays a key linkage role in ROS production by RANKL. Rac1 and NOX form the sequential order of the signaling cascade of ROS production. In this study, it was observed that RANKL stimulation led to a dose-dependent increase in ROS production. The levels of ROS rapidly reached their peak at approximately 15 min and subsequently declined after 30 min in the cytoplasm. Additionally, the levels of ROS in both the cytoplasm and the mitochondria increased after 24 and 48 h of RANKL treatment. Notably, the *Nrf2*-silenced group exhibited higher levels of ROS production compared to the scramble group in response to RANKL treatment. These findings provide clear evidence that *Nrf2* deficiency enhances RANKL-induced ROS production, highlighting the crucial role of Nrf2 in osteoclastogenesis.

NAC has been shown to significantly reduce ROS concentrations. DPI, a non-reversible inhibitor of flavoprotein, NOX, and xanthine oxidase, has also been found to decrease ROS levels. Additionally, MitoQ, a mitochondria-targeted antioxidant, has been demonstrated to inhibit the production of ROS within the mitochondria. The levels of intracellular ROS directly impact RANKL-induced osteoclast differentiation and bone density. Antioxidants such as NAC, DPI, and MitoQ effectively suppress osteoclastogenesis by scavenging ROS activity. In this study, it was observed that the rapid augmentation in ROS production induced by RANKL reached its maximum level at approximately 15 min. Pretreatment with NAC and DPI demonstrated significant inhibition of the transient rise in ROS levels caused by *Nrf2* deficiency in response to RANKL treatment. However, these pretreatments did not exhibit a significant inhibitory effect on the promotion of osteoclast differentiation caused by *Nrf2* deficiency. These findings suggest that the transient elevation of ROS resulting from RANKL treatment does not exert any influence on the process of osteoclast differentiation. The detection of ROS levels was conducted using DCFH-DA and MitoSOX. The results revealed increased ROS levels in both the cytoplasm and mitochondria following 48 h of RANKL treatment. To investigate the impact of antioxidants (NAC, DPI, and MitoQ) on osteoclast differentiation, the cells were treated with these compounds for the first two days and throughout the differentiation process. It was observed that NAC, DPI, and MitoQ effectively inhibited osteoclast differentiation. These findings suggest that *Nrf2* deficiency enhances the accumulation of RANKL-induced oxidative stress during osteoclastogenesis, thereby promoting osteoclast differentiation. Hence, the utilization of Nrf2 as a therapeutic target to impede osteoclast differentiation can be achieved through the activation of Nrf2 and the augmentation of intracellular antioxidant enzymes, while concurrently suppressing intracellular ROS levels.

c-FOS belongs to the FOS gene family, including c-FOS, FosB, Fra-1, and Fra-2, which is a component of the AP-1 family of transcriptional activators. RANKL binds to RANK, resulting in the recruitment of TRAF6 in the cytoplasm, then triggers the downstream NF-κB and MAPK signaling, which subsequently activates transcription factors c-FOS and NFATc1. This enhances the expression of osteoclastogenesis-related markers, eventually leading to the differentiation and maturation of osteoclasts [37]. The RANKL signaling pathway has the potential to induce the activation of the c-FOS signaling pathway through the elevation of intracellular ROS levels, thereby facilitating the process of osteoclast differentiation. Further investigation is warranted to elucidate the precise influence of intracellular ROS levels on c-FOS expression during osteoclast differentiation, as well as the role of Nrf2 in this process. In the preceding findings of this investigation, it was observed that the deficiency of *Nrf2* had a more pronounced impact on the elevation of ROS levels induced by RNAKL treatment. Additionally, the protein levels of c-FOS generated during differentiation were significantly inhibited by NAC treatment in *Nrf2*-deficient cells. The results of this investigation indicate that the lack of *Nrf2* may exert an influence on osteoclast differentiation by increasing the levels of ROS in both the cytoplasm and mitochondria, thereby affecting the expression of c-FOS within the cells. To further investigate the role of c-FOS in osteoclast differentiation, we conducted experiments involving the silencing of *c-Fos* and *Nrf2* to induce this process. Our findings indicate that the absence of *c-Fos* hinders the promotion of osteoclast differentiation caused by *Nrf2* deletion. Additionally, the compound T5224, known for its specific inhibition of c-FOS, effectively impedes the increase in osteoclast differentiation that results from *Nrf2* deletion. The presented data indicate that c-FOS significantly contributes to the promotion of osteoclast differentiation in cells lacking *Nrf2*, thereby suggesting that c-FOS serves as a prominent downstream target of Nrf2 in osteoclasts.

Our current study has several limitations. Firstly, osteoclast differentiation involves multiple signaling pathways, including NF-κB and MAPK. Therefore, it is necessary to conduct further analysis on the impact of increased ROS levels resulting from *Nrf2* deficiency on these signaling pathways at different stages of differentiation. Secondly, our study primarily relied on in vitro experiments, and additional evidence from in vivo experiments is required in order to gain a deeper understanding of the role of Nrf2 in osteoclast differentiation. Furthermore, it is imperative to identify specific activators of Nrf2 in forthcoming research endeavors, as this will establish a theoretical framework for the prevention and treatment of osteoporosis.

## 5. Conclusions

In conclusion, our study presents empirical evidence suggesting a decline in the expression levels of Nrf2 and its associated antioxidant genes throughout the osteoclast differentiation process, concomitant with an elevation in ROS levels within the cytoplasm and mitochondria. Furthermore, our study provides evidence that the absence of *Nrf2* can augment the process of osteoclast differentiation through the upregulation of ROS levels within the cytoplasm and the mitochondria. Additionally, our research indicates that the administration of antioxidants successfully inhibits the intracellular levels of ROS and mitigates the increased differentiation of osteoclasts caused by the deletion of *Nrf2*. Altogether, we have uncovered the central role of Nrf2 in osteoclastogenesis. Our findings contribute to a better understanding of the underlying pathways by which Nrf2 exerts a negative regulatory effect on osteoclastogenesis, primarily by inhibiting the production of ROS and suppressing the expression of c-FOS.

## Data Availability

Data are contained within the article.

## Supplementary Materials

The following supporting information can be downloaded at: https://www.mdpi.com/article/10.3390/antiox12122094/s1, Figure S1. Overexpression of Nrf2 inhibits differentiation of osteoclasts in RAW 264.7 cells. Figure S2. Nrf2 expression and its downstream genes exhibited a decrease during osteoclastogenesis. Figure S3. Deficiency of *Nrf2* is more sensitive to transient RANKL-induced cytoplasmic ROS generation. Figure S4. Transient RANKL-treatment did not yield an increase in reactive oxygen species within the mitochondria. Figure S5. Antioxidant pretreatment had no effect on the enhancement of osteoclastogenesis caused by *Nrf2* deficiency. Figure S6. NAC treatment inhibits osteoclast differentiation induced by *Nrf2-*deficiency. Figure S7. DPI treatment inhibits osteoclast differentiation induced by *Nrf2*-deficiency. Figure S8. MitoQ treatment inhibits osteoclast differentiation induced by *Nrf2-*deficiency. Figure S9. Successful establishment of *c-Fos knockdown* in RAW 264.7. mRNA levels of *c-Fos* in RAW 264.7. Figure S10. T5224 suppresses the expression of c-FOS and NFATc1. Table S1. The gene accession number employed in this article.

## Abbreviations

ATCC: American Type Culture Collection; α-MEM: minimum essential medium alpha medium; BMMs: bone-marrow-derived macrophages; DCFH-DA: 2′,7′-dichlorofluorescein diacetate; DMEM: Dulbecco’s modified essential media; DPI: diphenyleneiodonium chloride; Ho-1: heme oxygenase-1; HI-FBS: heat-inactivated fetal bovine serum; *Gclc*: glutamate cysteine ligase catalytic subunit; *Gclm*: glutamate cysteine ligase modifier subunit; *Keap1*: Kelch-like ECH-associated protein-1; M-CSF: macrophage colony-stimulating factor; MitoQ: mitoquinone mesylate; MW: molecular weight; NAC: N-acetylcysteine; NFATc1: nuclear factor of activated T cells, cytoplasmic, calcineurin-dependent 1; NOX1: NADPH oxidase 1; *Nqo1*: NAD(P)H quinine oxidoreductase-1; Nrf2: nuclear factor erythroid-derived 2-like 2; *Nrf2*^−/−^: Global *Nrf2* knockout; *Nrf2^+/−^*: *Nrf2-*heterozygous; *Nrf2*^+/+^: *Nrf2*-wildtype; *Nrf2*-KD: *Nrf2* knockdown; *Nrf2*-OE: *Nrf2* overexpress mouse; OPCs: osteoclast progenitor cells; PBS: phosphate-buffered saline; PMSF: phenylmethylsulfonyl fluoride; PVDF: polyvinylidene difluoride; RANKL: receptor activator of nuclear factor-κB ligand; ROS: reactive oxygen species; Scr: scramble; SD: standard deviation; TBS: Tris-buffered saline; TRAP: tartrate-resistant acid phosphatase; TRAF6: tumor necrosis factor receptor-associated factor 6.
