# Supplementary material for: Nrf2 Mitigates RANKL and M-CSF Induced Osteoclast Differentiation via ROS-Dependent Mechanisms
Source: Antioxidants (Basel). 2023 Dec 10;12(12):2094. doi: 10.3390/antiox12122094 (PMC10740485; doi:10.3390/antiox12122094)
Supplement: Supplementary file 1 [file antioxidants-12-02094-s001.zip › antioxidants-2694089-supplementary.pdf]

Supplemental information

Nrf2 mitigates RANKL and M-CSF induced osteoclast differentiation via ROS-dependent mechanisms

Yang Yang, Zhiyuan Liu, Jinzhi Wu, Simeng Bao, Yanshuai Wang, Jiliang Li, Tao Song, Yongxin Sun, Jingbo Pi

Corresponding author: J.P., jbpi@cmu.edu.cn, jingbopi@163.com;

Y.S., sunyongxin@cmu.edu.cn.

Name	Gene Accession Number
Atp6v0d2	NM_175406
Cathepsin K	NM_007802
c-FOS	NM_010234
c-JUN	NM_010591
Gclc	NM_010295
Gclm	NM_008129
H^+-ATPase	NM_001081356
HO-1	NM_010442
Keap1	NM_016679
NFATc1	NM_001164112
Nqo1	NM_008706
Nrf2	NM_010902
Oscar	NM_175632
RANKL	NM_011613
RANK	NM_009399
TRAF6	NM_001303273
β-ACTIN	NM_001289726

Table S1. The gene accession number employed in this article.

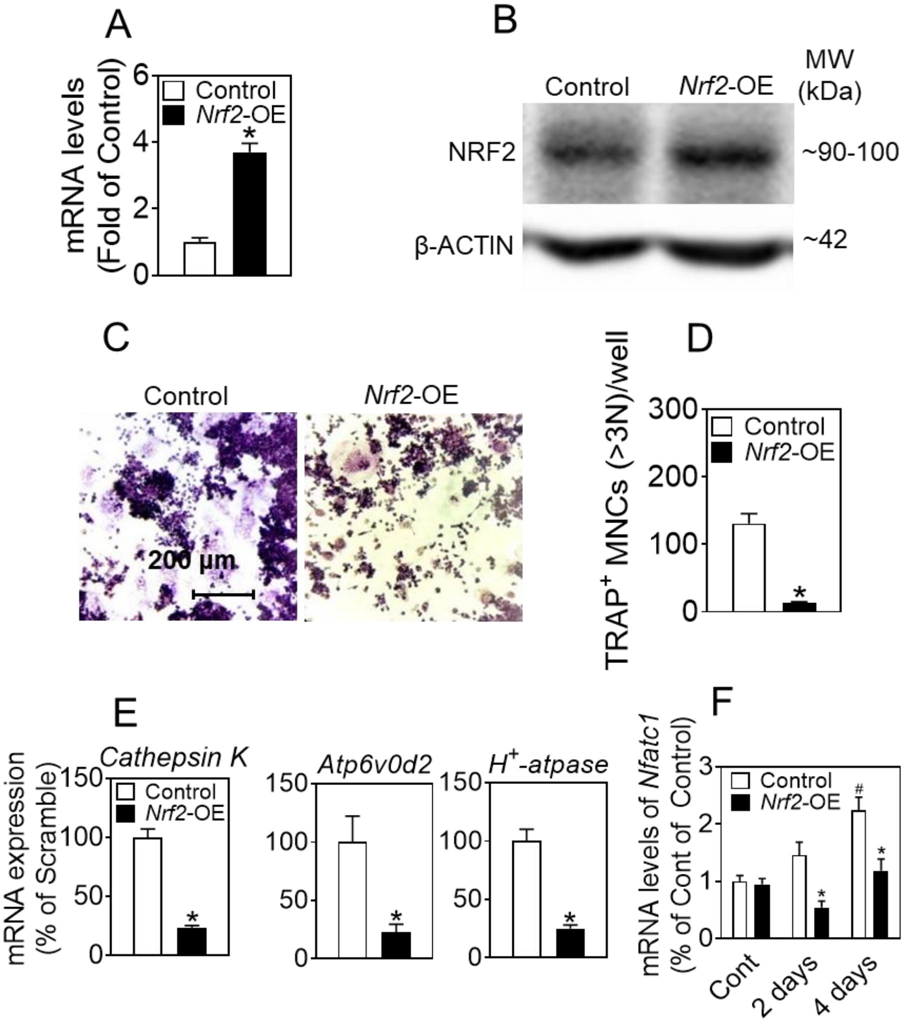

Figure S1. Overexpression of Nrf2 inhibits differentiation of osteoclasts in RAW 264.7 cells. (A) mRNA level of *Nrf2* in RAW 264.7 cells. **(**B**)** Protein level of NRF2. (C and D) TRAP staining and quantification of TRAP-positive multinucleated cells containing more than 3 nuclei in *Nrf2*-OE RAW 264.7 cells. (E) mRNA levels of *Cathepsin K*, *Atp6v0d2* and *H^+-Atpase* in *Nrf2*-OE RAW 264.7 cells. Values are mean ± SD. n = 3, Student's t test was used, *$p < 0.05$ vs Control. Scale bars = 200 μm. (F) mRNA levels of *Nfatc1* in *Nrf2*-OE RAW 264.7 cells with treatment of RANKL and M-CSF for Cont, 2 and 4 days. Values are mean ± SD. n = 3, two-way ANOVA was used, *$p < 0.05$ vs Control with the same treatment, #$p < 0.05$ vs Control of the same cytotype.

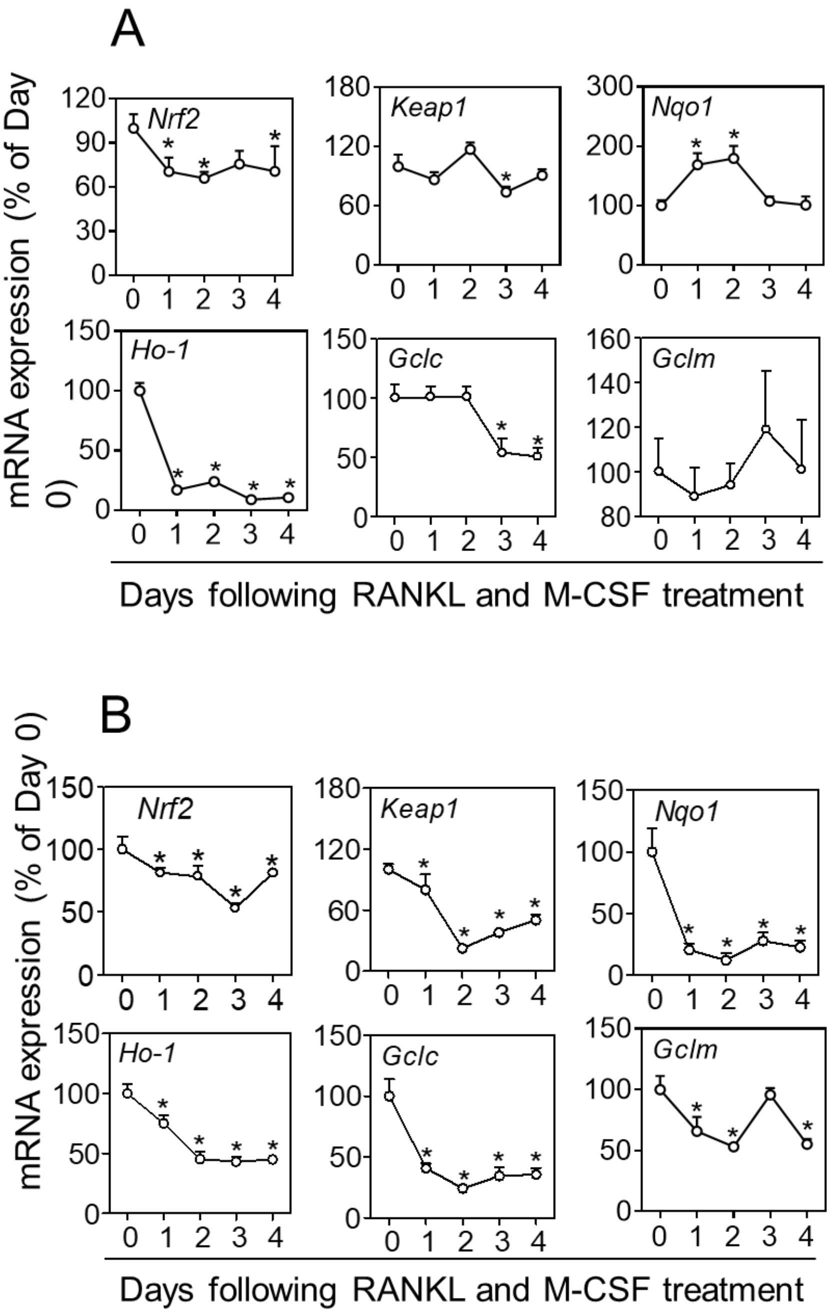

Figure S2. Nrf2 expression and its downstream genes exhibited a decrease during osteoclastogenesis.

(A and B) BMMs and RAW 264.7 cells were treated with 50 ng/mL RANKL and 30 ng/mL for 1, 2, 3 and 4 days. mRNA levels of *Nrf2*, *Keap1*, *Nqo1*, *Ho-1*, *Gclc* and *Gclm* were isolated from BMMs (A) and RAW 264.7 cells (B). Values are mean ± SD. n = 3, one-way ANOVA was used, *$p < 0.05$ vs 0 day.

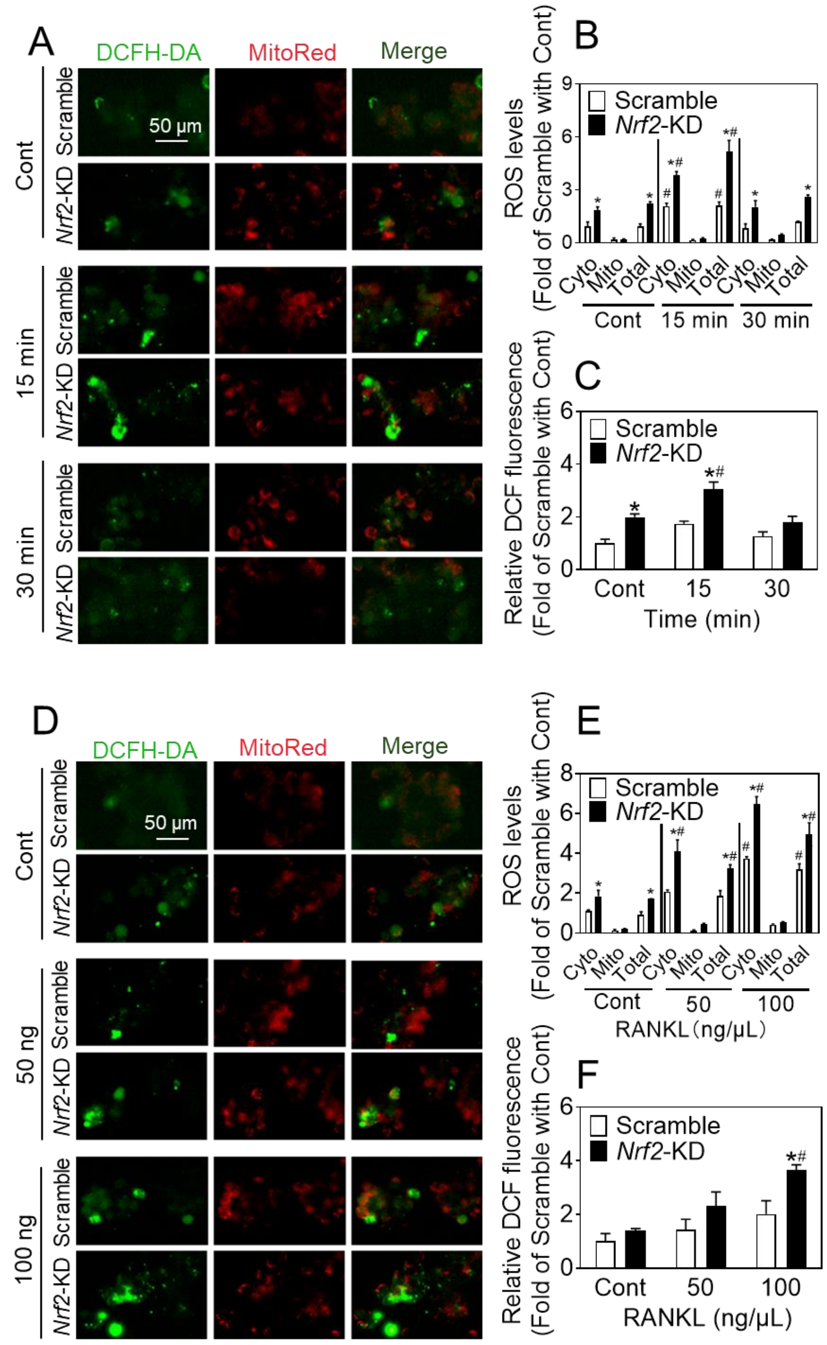

Figure S3. Deficiency of *Nrf2* is more sensitive to transient RANKL-induced cytoplasmic ROS generation.

Nrf2-KD RAW 264.7 cells were preloaded with DCFH-DA and then treated with 100 ng/mL RANKL for 15 and 30 minutes. Representative images (A) and quantification (B) of ROS levels measured by fluorescence microscope. Scale bars: 50 μm. ROS levels were measured by flow cytometry (C). *Nrf2*-KD RAW 264.7 cells were preloaded with DCFH-DA and then treated with 50 and 100 ng/mL RANKL for 15 minutes. Representative images and quantification of ROS levels measured by fluorescence microscope (D-E). ROS levels were measured by flow cytometry (F). Scale bars: 50 μm. Values are mean ± SD. n = 3, two-way ANOVA was used, $^{*}p < 0.05$ vs Scramble with the same treatment, $^{\#}p < 0.05$ vs Control of the same cytotype.

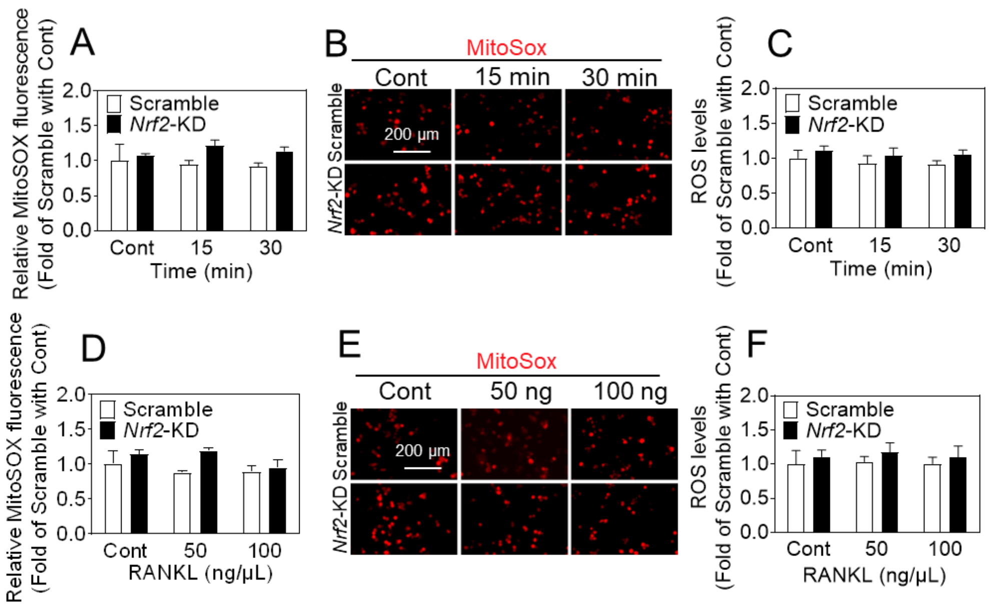

Figure S4. Transient RANKL-treatment did not yield an increase in reactive oxygen species within the mitochondria.

Nrf2-KD RAW 264.7 cells were preloaded with MitoSox for 30 minutes and then treated with 100 ng/mL RANKL for 15 and 30 minutes. (A) ROS levels were measured by flow cytometry. Representative images and quantification of ROS levels measured by fluorescence microscope (B and C). *Nrf2*-KD RAW 264.7 cells were preloaded with MitoSox for 30 minutes and then treated with 50 and 100 ng/mL RANKL for 15 minutes. (D) ROS levels were measured by flow cytometry. (E-F) Representative images and quantification of ROS levels measured by fluorescence microscope. Scale bars: 50 μm. Values are mean ± SD. n = 3, two-way ANOVA was used.

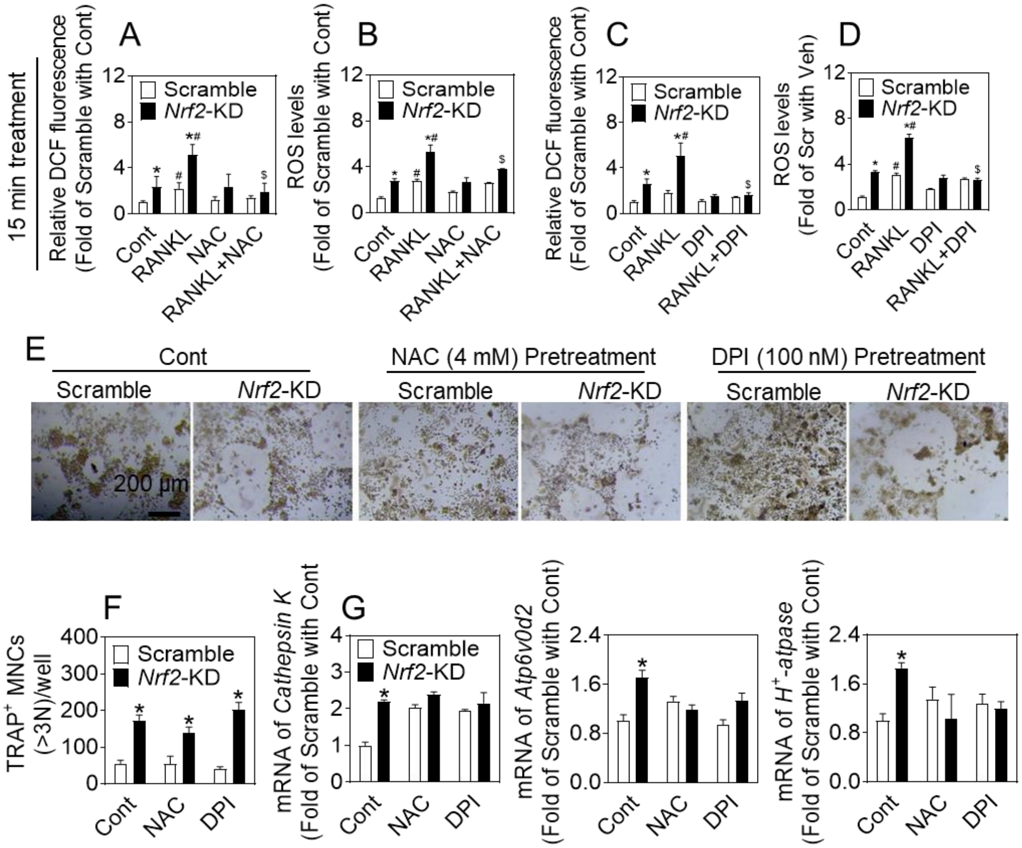

Figure S5. Antioxidant pretreatment had no effect on the enhancement of osteoclastogenesis caused by *Nrf2* deficiency.

Nrf2-KD RAW 264.7 cells were pretreated with NAC (4 mM) or DPI (50 nM) and then treated with 100 ng/mL RANKL for 15 minutes. ROS levels were measured by flow cytometry (A and C). Quantification (B and D) of ROS levels measured by fluorescence microscope. (E) TRAP staining of RAW 264.7 cells were treated with RANKL (50 ng/mL) and M-CSF (30 ng/mL) for 4 days with or without of NAC (4 mM) or DPI (50 nM) pretreatment. (F) Quantification of TRAP-positive multinucleated cells containing more than 3 nuclei. (G) mRNA levels of *Cathepsin K*, *Atp6v0d2* and *H^+-Atpase* in *Nrf2*-KD RAW 264.7 cells pretreated with NAC or DPI. Scale bars: 200 μm. Values are mean ± SD. n = 3, *$p < 0.05$ vs Scramble with the same treatment. $^{\#}p < 0.05$ vs Control of the same cytotype, $^{\$}p < 0.05$ vs RANKL treatment of the same cytotype.

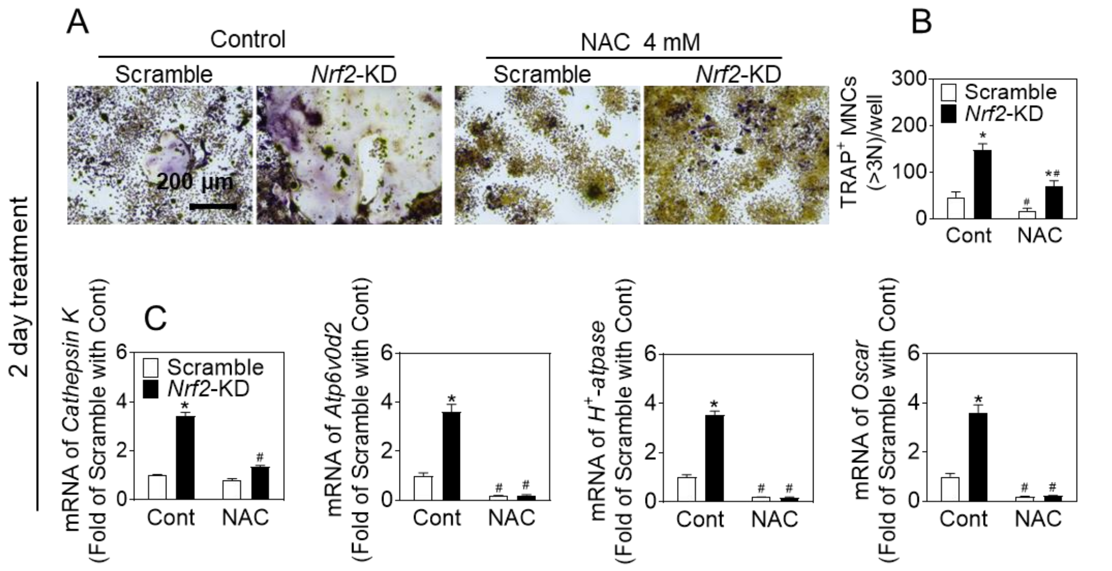

Figure S6. NAC treatment inhibits osteoclast differentiation induced by *Nrf2*-deficiency.
(A) TRAP staining of RAW 264.7 cells treated with RANKL (50 ng/mL) and M-CSF (30 ng/mL) for 2 days with or without of NAC (4 mM). (B) Quantification of TRAP-positive multinucleated cells containing more than 3 nuclei. Scale bars = 200 μm. (C) mRNA levels of *Cathepsin K*, *Atp6v0d2* and H^{+}-*Atpase* in *Nrf2*-KD RAW 264.7 cells treated with RANKL (50 ng/ml) and M-CSF (30 ng/mL) for 2 days with or without of NAC (4 mM). Values are mean ± SD. n = 3, two-way ANOVA was used, *$p < 0.05$ vs Scramble with the same treatment, $^{\#}p < 0.05$ vs Control of the same cytotype.

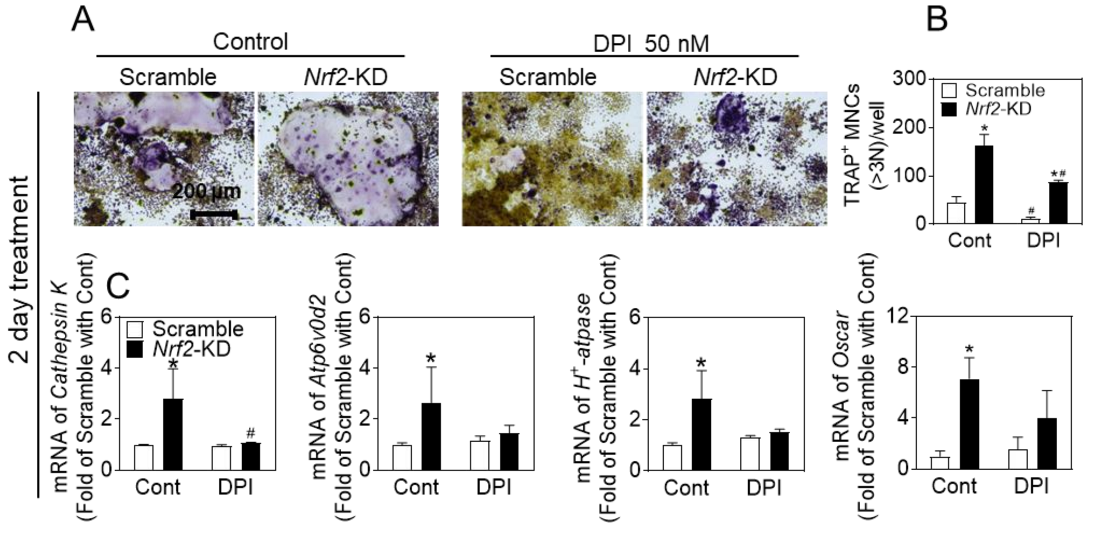

Figure S7. DPI treatment inhibits osteoclast differentiation induced by *Nrf2*-deficiency.
(A) TRAP staining of RAW 264.7 cells treated with RANKL (50 ng/mL) and M-CSF (30 ng/mL) for 2 days with or without of DPI (50 nM). (B) Quantification of TRAP-positive multinucleated cells containing more than 3 nuclei. Scale bars = 200 μm. (C) mRNA levels of *Cathepsin K*, *Atp6v0d2* and H^{+}-*Atpase* in *Nrf2*-KD RAW 264.7 cells treated with RANKL (50 ng/mL) and M-CSF (30 ng/mL) for 2 days with or without of DPI (50 nM). Values are mean ± SD. n = 3, two-way ANOVA was used, *$p < 0.05$ vs Scramble with the same treatment, $^{\#}p < 0.05$ vs Control of the same cytotype.

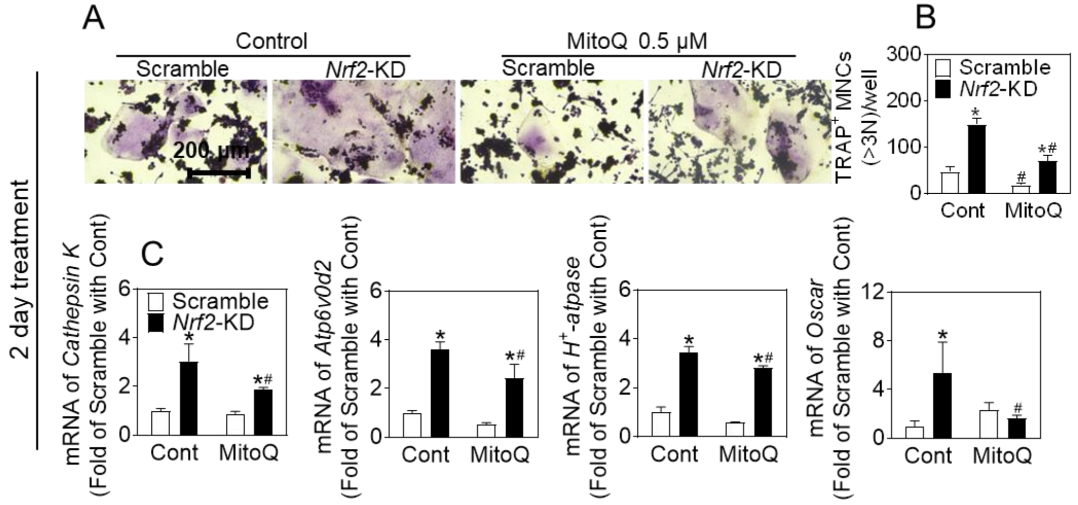

Figure S8. MitoQ treatment inhibits osteoclast differentiation induced by *Nrf2*-deficiency.
(A) TRAP staining of RAW 264.7 cells treated with RANKL (50 ng/mL) and M-CSF (30 ng/mL) for 2 days with or without of MitoQ (0.5 μM). (B) Quantification of TRAP-positive multinucleated cells containing more than 3 nuclei. Scale bars = 200 μm. (C) mRNA levels of *Cathepsin K*, *Atp6v0d2* and *H^+-Atpase* in *Nrf2*-KD RAW 264.7 cells treated with RANKL (50 ng/mL) and M-CSF (30 ng/mL) for 2 days with or without of MitoQ (0.5 μM). Values are mean ± SD. n = 3, two-way ANOVA was used, **p* < 0.05 vs Scramble with the same treatment. $^{\#}p < 0.05$ vs Control of the same cytotype.

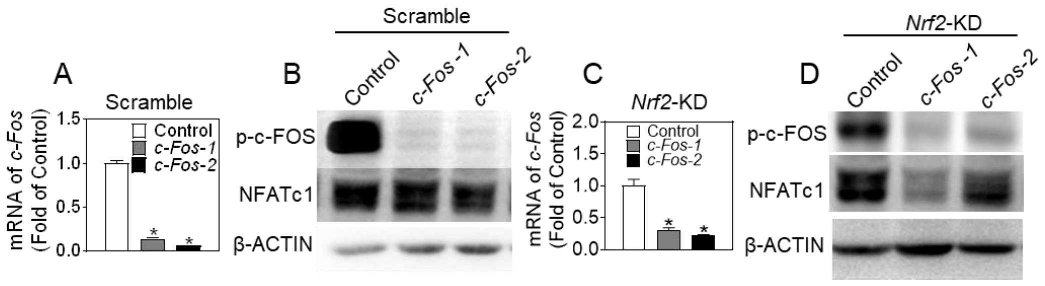

Figure S9. Successful establishment of *c-Fos* knockdown in RAW 264.7. mRNA levels of *c-Fos* in RAW 264.7.
Scramble and *Nrf2*-KD cells transduced with shRNA against *c-Fos*, mRNA levels of *c-Fos* in scramble (A) and *Nrf2*-KD (C) RAW 264.7 cells. Values are mean ±SD. n = 3, one-way ANOVA was used, **p* < 0.05 vs Control. Protein levels of p-c-FOS and NFATc1 in Scramble (B) and *Nrf2*-KD (D) RAW 264.7 cells.

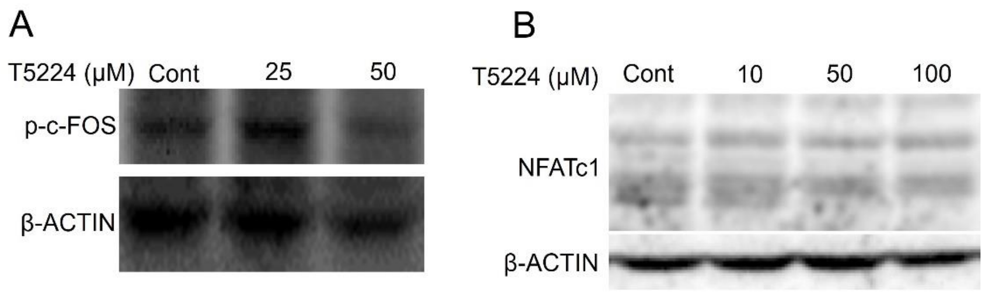

Figure S10. T5224 suppresses the expression of c-FOS and NFATc1.
(A and B) RAW 264.7 cells were treated with different concentrations of T5224 for 24 hours, and protein levels of p-c-FOS and NFATc1 were determined by western blot.
